# Integrating kidney imaging for risk prediction, therapeutic monitoring, and prognostication across the kidney disease spectrum: a review of emerging evidence

**DOI:** 10.1093/ckj/sfag243

**Published:** 2026-08-04

**Authors:** Mustafa Guldan, Ibrahim Gulmaliyev, Rama AlShiab, Ermeena Shah, Lasin Ozbek, Mahmut Altindal, Bengi Gurses, Magdalena Madero, Alberto Ortiz, Adrian Covic, Mehmet Kanbay

**Affiliations:** Department of Medicine, Koc University School of Medicine, Istanbul, Turkiye; Department of Medicine, Koc University School of Medicine, Istanbul, Turkiye; Department of Medicine, Koc University School of Medicine, Istanbul, Turkiye; Department of Medicine, Koc University School of Medicine, Istanbul, Turkiye; Department of Medicine, Koc University School of Medicine, Istanbul, Turkiye; Department of Internal Medicine, Division of Nephrology, Koc University School of Medicine, Istanbul, Turkiye; Department of Radiology, Koc University School of Medicine, Istanbul, Turkiye; Department of Nephrology, Instituto Nacional de Cardiología Ignacio Chavez, Mexico City, Mexico; Department of Medicine, Universidad Autonoma de Madrid and IIS-Fundacion Jimenez Diaz, Madrid, Spain; Nephrology Clinic, Dialysis and Renal Transplant Center, “C.I. Parhon” University Hospital and “Grigore T. Popa” University of Medicine, Iasi, Romania; Department of Internal Medicine, Division of Nephrology, Koc University School of Medicine, Istanbul, Turkiye

**Keywords:** artificial intelligence, chronic kidney disease, diabetic kidney disease, kidney transplantation, multiparametric MRI, radiomics, renal fibrosis, renal imaging, risk prediction, therapeutic monitoring, ultrasound elastography

## Abstract

Kidney imaging advances are transforming the field of nephrology by allowing non-invasive examination of renal structure, function, and pathology. Traditional measures such as albuminuria and estimated glomerular filtration rate (eGFR) are only partially effective in identifying early or regionally variable kidney damage. As an example, imaging identifies chronic kidney disease (CKD) in autosomal dominant polycystic kidney disease (ADPKD) decades earlier than eGFR or albuminuria, addressing the “blind spot” in CKD, and providing a criterion to start early therapy. Currently, quantitative imaging techniques such as advanced ultrasound (Doppler sonography, photoacoustic imaging [PAI], contrast-enhanced ultrasound [CEUS], and ultrasound-based elastography), multiparametric MRI, and some CT methods provide insights into fibrosis, lipid infiltration, oxygenation, and microvascular integrity. Imaging biomarkers, including cortical *R*_2_*, apparent diffusion coefficient (ADC), arterial spin labeling (ASL), and shear-wave elastography (SWE), show significant predictive value for progression to kidney failure in CKD, including diabetic kidney disease, independent of traditional measurements. Imaging detects fibrotic remodeling and microvascular impairment in hypertensive and obese kidney phenotypes before laboratory indicators appear. Emerging technologies such as AI-augmented radiomics, photoacoustic oxygen mapping, and H-scan ultrasound may facilitate the early detection of graft fibrosis and functional decline in kidney transplantation. Most methods, however, lack optimal clinical integration, standardized protocols, and comprehensive validation. Future directions may include longitudinal cohort studies, multi-omics data integration, imaging-informed clinical trial outcomes, and machine learning-based prognostic models. Imaging biomarkers have the potential to profoundly transform precision nephrology by providing organ- and tissue-level insights that enhance transplant surveillance, therapy monitoring, and personalized risk classification.

## INTRODUCTION

Around 850 million people globally have chronic kidney disease (CKD), a serious public health issue that raises morbidity, mortality, and medical expenses [1]. High-quality nephrology care rests on three pillars: prompt detection, accurate risk assessment, and individualized treatment regimens [2]. For assessing the activity and progression of the disease, standard clinical indicators such as albuminuria and estimated glomerular filtration rate (eGFR) are insufficient and late indicators. The measurements indicate considerable biological variability, inadequate spatial resolution, and insufficient characterization of the microstructural and functional alterations related to kidney injury [3, 4]. The CKD blind spot phenomenon is illustrated by the ability of imaging to diagnose CKD in patients with autosomal dominant polycystic kidney disease (ADPKD) decades earlier than eGFR or albuminuria thresholds, enabling the initiation of specific treatment [5–7]. This supports the biological plausibility that imaging advances can enable earlier, proactive management of CKD, potentially preserving kidney health [8].

Recent advancements in quantitative imaging have markedly improved the assessment of kidney disease. Advanced ultrasound (US) techniques, such as US elastography and contrast-enhanced Doppler, and multiparametric magnetic resonance imaging (MRI) enable the noninvasive evaluation of renal perfusion, fibrosis, oxygenation, lipid accumulation, and microvascular integrity, providing improved spatial and temporal accuracy [9, 10]. These modalities may surpass conventional laboratory assays and kidney biopsy in identifying early, subclinical changes that occur prior to significant functional decline, categorizing patients by progression risk, and facilitating real-time therapeutic monitoring [3, 10]. While biopsy remains the reference standard for histologic assessment, imaging offers a complementary, non-invasive view of both kidneys and can detect spatial heterogeneity beyond the limited sampling inherent to biopsy [11].

The integration of radiomics, artificial intelligence (AI), and multimodal data fusion enhances the diagnostic and prognostic capabilities of renal imaging, especially in the areas of transplant nephrology and CKD [12, 13]. This review synthesizes recent evidence on the role of renal imaging in risk stratification, therapeutic monitoring, and prognostic evaluation across kidney diseases. We highlight applications in CKD, its major causes [diabetic kidney disease (DKD) and hypertensive nephrosclerosis], and kidney transplantation, and explore integration with molecular biomarkers, AI, and omics-based tools. Future directions involve establishing imaging as a cornerstone of precision nephrology through clinical trials, multimodal approaches, and integration with molecular biomarkers, AI, and omics-based tools.

**Figure 1:**
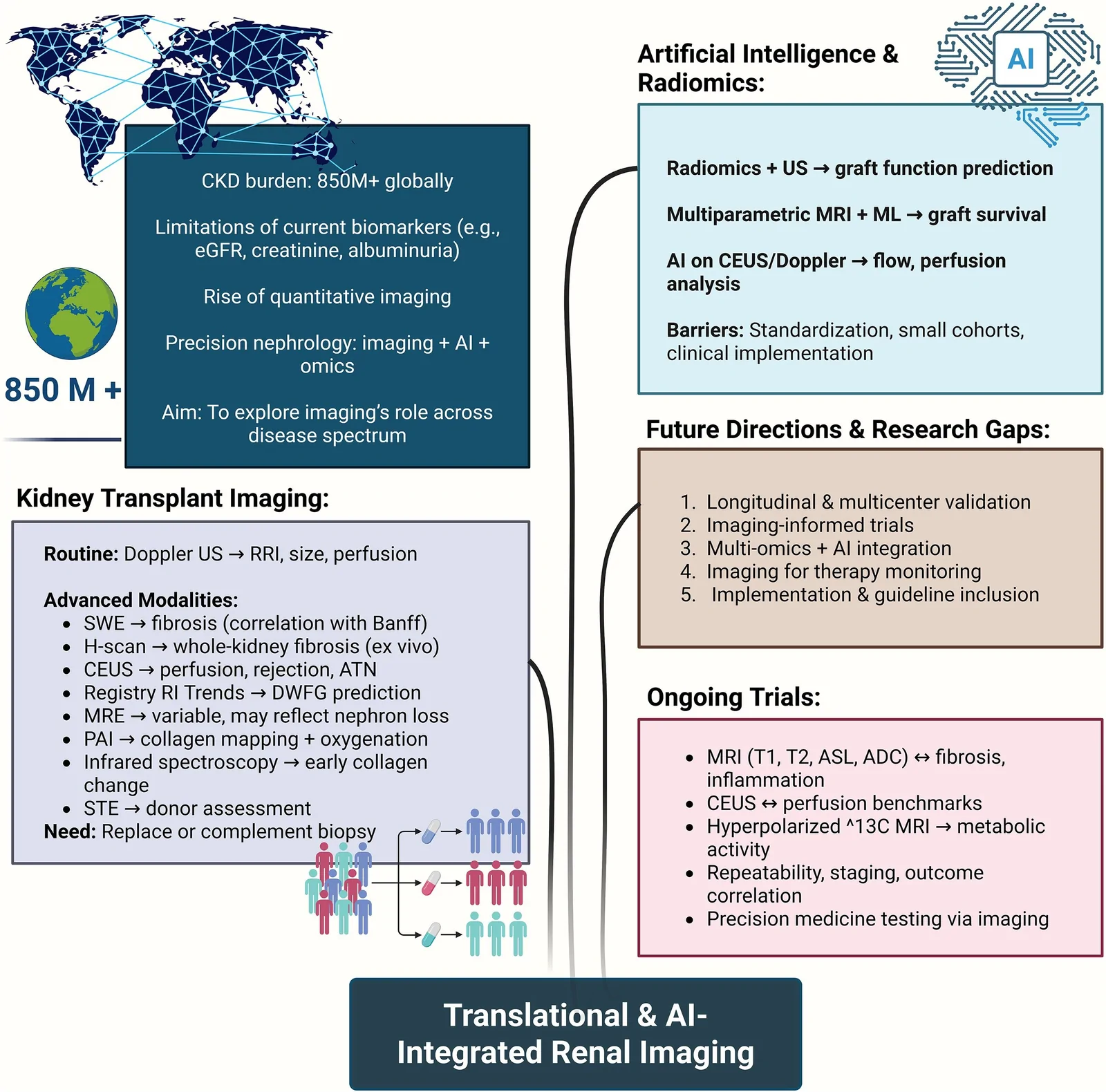
Quantitative and functional renal imaging across the CKD spectrum. Quantitative MRI and ultrasound modalities enable early detection of key pathophysiological changes—fibrosis, hypoxia, and microvascular rarefaction—before traditional biomarkers (eGFR, albuminuria) decline. Techniques, such as, BOLD MRI, ASL, IVIM-DWI, T1 mapping, and elastography characterize oxygenation, perfusion, and tissue stiffness across chronic, diabetic, and metabolic kidney diseases. Early imaging-based detection and risk stratification may facilitate timely therapeutic action, slowing or halting CKD progression. **Abbreviations:** ADC, apparent diffusion coefficient; AI, artificial intelligence; ARFI, acoustic radiation force impulse; ASL, arterial spin labeling; BOLD, blood oxygenation level-dependent; CEUS, contrast-enhanced ultrasound; CKD, chronic kidney disease; CVD, cardiovascular disease; DKD, diabetic kidney disease; DWFG, death with functioning graft; eGFR, estimated glomerular filtration rate; IVIM-DWI , intravoxel incoherent motion diffusion-weighted imaging; MRA. magnetic resonance angiography; MRE. magnetic resonance elastography; MRI. magnetic resonance imaging; MRS. magnetic resonance spectroscopy; PAI. photoacoustic imaging; PDFF. proton-density fat fraction; PRS. polygenic risk score; *R*_2_*. transverse relaxation rate; RI. resistive index; RRI, renal resistive index; RSF, renal sinus fat; SMI, superb microvascular imaging; SWE, shear-wave elastography; STE, strain-tensor elastography; T1/T2, relaxation times; and US, ultrasound.

Specific features of renal imaging, such as multiparametric MRI in CKD, sophisticated US techniques, and the use of radiomics and AI in KT or CKD, have lately been covered in a number of authoritative reviews and position papers. Although these studies have yielded valuable insights related to modality or disease, they primarily take imaging into account in isolated clinical settings or as a technological supplement [3, 9, 13]. The current review, on the other hand, purposefully takes a cross-disease approach, incorporating data from early kidney injury through advanced CKD and KT, and placing more emphasis on quantitative imaging biomarkers with proven prognostic and therapeutic relevance than just descriptive imaging features (Fig. 1). This review attempts to provide a unified, translational roadmap for how renal imaging can inform risk prediction, therapeutic monitoring, and personalized care across the spectrum of kidney disease by synthesizing biomarkers of fibrosis, perfusion, oxygenation, microvascular integrity, and renal adiposity derived from ultrasound and MRI across disease states and placing these markers within emerging AI-enabled and multimodal precision-nephrology frameworks. Targeted searches of PubMed, Embase, and Web of Science were used to inform this narrative review, which mostly focused on research published in the last 10–15 years. Combinations of “chronic kidney disease,” “diabetic kidney disease,” “kidney transplantation,” “ultrasound elastography,” “contrast-enhanced ultrasound,” “BOLD MRI,” “arterial spin labeling,” “diffusion-weighted imaging,” “radiomics,” “renal fibrosis,” and “fatty kidney” were among the search terms used. Human studies, prospective cohorts, meta-analyses, and studies showing therapeutic or prognostic relationships were prioritized, with some preclinical data included where mechanistically instructive.

## IMAGING IN EARLY RENAL INJURY

eGFR and albuminuria remain indispensable for the diagnosis, risk stratification, and monitoring of CKD, as clinical trials have used both eGFR and albuminuria as entry criteria and outcomes [14]. However, they oscillate during the day and from day to day, reveal little about the micro-environmental injury driving long-term decline and are late events: by the time CKD is diagnosed based on eGFR, around 50% of the functional kidney mass has been lost, and the albuminuria threshold is 10-fold higher than physiological levels. Histologically, low-grade fibrosis, microvascular rarefaction, and cortical hypoxia are established early in the disease course [3]. In fatty kidney, ectopic lipid accumulated in three main compartments of the kidney: the perirenal space, the renal sinus and, less frequently, the cortex and medulla, leading to tubular inflammation and fibrosis, while fat deposited in the renal sinus can compress low-pressure veins and lymphatics, increasing interstitial pressure and local hypoxia [15–17].

**Figure 2:**
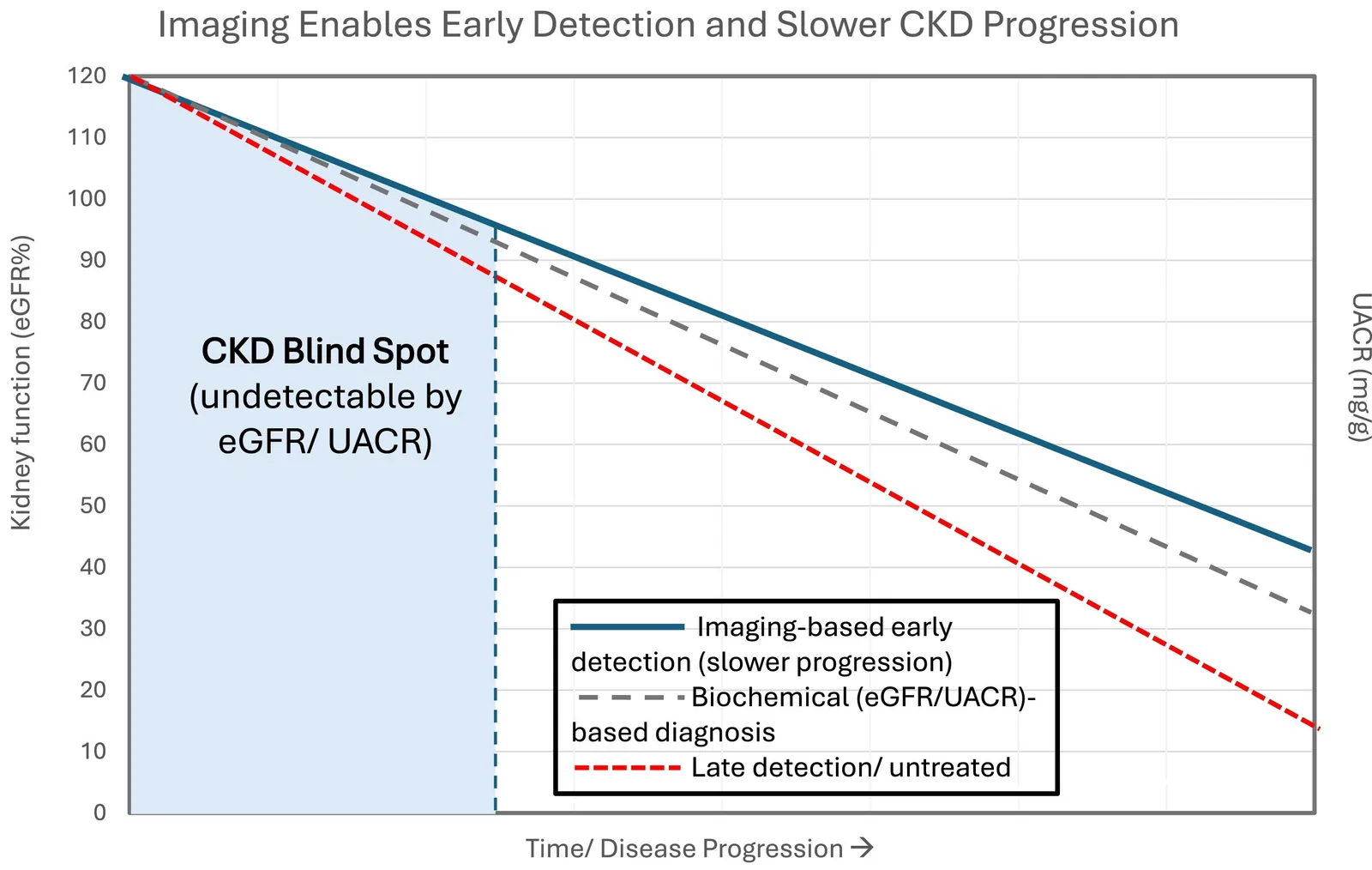
Translational and AI-integrated renal imaging: from quantitative biomarkers to clinical application. Advanced renal imaging extends beyond structure to function and prognosis. In hypertension and kidney transplantation, SWE, CEUS, and MRE assess fibrosis, perfusion, and rejection noninvasively, while AI and radiomics enhance feature extraction and graft outcome prediction. Integration with multi-omics approaches supports precision nephrology. Future priorities include longitudinal validation, imaging-informed clinical trials, and inclusion of imaging biomarkers in therapeutic decision-making. **Abbreviations:** ADC, apparent diffusion coefficient; AI, artificial intelligence; ARFI, acoustic radiation force impulse; ASL, arterial spin labeling; BOLD, blood oxygenation level-dependent; CEUS, contrast-enhanced ultrasound; CKD, chronic kidney disease; CVD, cardiovascular disease; DKD, diabetic kidney disease; DWFG, death with functioning graft; eGFR, estimated glomerular filtration rate; IVIM-DWI, intravoxel incoherent motion diffusion-weighted imaging; MRA, magnetic resonance angiography; MRE, magnetic resonance elastography; MRI, magnetic resonance imaging; MRS, magnetic resonance spectroscopy; PAI, photoacoustic imaging; PDFF, proton-density fat fraction; PRS, polygenic risk score; *R*_2_*, transverse relaxation rate; RI, resistive index; RRI, renal resistive index; RSF, renal sinus fat; SMI, superb microvascular imaging; SWE, shear-wave elastography; STE, strain-tensor elastography; T1/T2, relaxation times; and US, ultrasound.

Advanced imaging techniques now allow these subclinical changes to be detected and quantified, offering the promise of far earlier diagnosis and more precise staging than conventional biomarkers (Fig. 2) [7]. Contemporary imaging pipelines interrogate oxygenation, perfusion, microstructural integrity, steatosis, and stiffness—often within a single session—enabling a truly multiparametric approach. Modern quantitative imaging bridges this gap by interrogating oxygenation, perfusion, micro-structural integrity, and stiffness *in vivo*, thereby providing complementary—and at times superior—prognostic information to laboratory tests alone. The location of the kidney graft confers certain advantages for imaging (Table 1).

**Table 1:** Comparative overview of renal imaging modalities and level of evidence summary.

Imaging method	Purpose/comparison focus	Key quantitative findings	Clinical applications	Level of evidence
Contrast-Enhanced Ultrasound (CEUS)	Detect microvascular perfusion deficits vs. Doppler US	Hypertensive patients show ∼28% lower cortical perfusion than controls (1476 vs. 2062 AU; *P* < 0.001) and blunted flow reserve (34 % vs. 56 %)	Early CKD microcirculatory assessment, renal mass characterization, transplant perfusion	**Level II b** (prospective controlled human studies)
Doppler Resistive Index (RRI)	Correlate vascular resistance with renal prognosis	RRI > 0.70 predicts faster eGFR decline; each 0.01 ↑ = 4 % ↑ mortality hazard & 6 % ↑ dialysis risk; ACE I ↓ RRI 0.61→0.56 (24 mo)	Routine CKD, hypertension, transplant evaluation	**Level II a** (meta-analysis + cohort)
Superb Microvascular Imaging (SMI)	Compare to Doppler US and CEUS for low-flow detection	Visualizes 300–500 μm vessels; qualitative detection of microvascular rarefaction mirroring ASL MRI findings	CKD cortical perfusion mapping, tumor vascularity, post-treatment monitoring	**Level III b** (pilot observational studies)
Ultrasound Elastography (SWE/ARFI)	Compare stiffness vs. biopsy-proven fibrosis	Meta-analysis (1394 pts): AUC 0.87 mild, 0.78 moderate, 0.86 severe; in DKD 19 kPa → 93 % sens/86 % spec; ARFI 2.37 vs. 2.96 m/s (HTN)	Noninvasive fibrosis quantification in CKD, DKD, HTN nephropathy	**Level I a** (systematic review + multiple RCT-validated cohorts)
BOLD MRI (Oxygenation)	Evaluate tissue oxygenation vs. eGFR/creatinine	Higher *R*₂* = 3 × risk ESKD; Dapagliflozin ↓ *R*₂* by 9 % (6 h) → ↑ oxygenation	Risk stratification, drug response (esp. SGLT2i effects)	**Level II a** (prospective cohort + intervention)
ASL MRI (Perfusion)	Quantify RBF vs. CKD stage	Threshold 143 ml/min/100 g distinguishes CKD (AUC 0.98); *r* ≈ 0.8 with eGFR	Quantitative non-contrast perfusion mapping for CKD & DKD	**Level II a** (prospective cohort + intervention)
IVIM-DWI (Diffusion)	Microstructure + microvascular flow vs. biopsy/fibrosis	Each 1 % ↓ in *f* = 21 % ↑ ESKD risk; AUC 0.99 for early DKD with D + FA combo	Structural injury marker for CKD progression prediction	**Level II b** (prospective controlled human studies)
T₁ Mapping (Fibrosis)	Quantify fibrosis vs. histology & outcomes	Highest *T*₁ = 3 × risk ESKD; radiomics AUC 0.93–0.94 for fibrosis >25 %	Non-contrast fibrosis biomarker for CKD/DKD monitoring	**Level II a** (prospective cohort + intervention)
Multiparametric MRI (mpMRI)	Integrate perfusion + oxygenation + diffusion + *T*₁	4-parameter model → only BOLD R₂* remains independent predictor of eGFR decline; repeatability CV ≈ 4%–11%	Comprehensive risk profiling in CKD and transplant	**Level II b** (prospective controlled human studies)
Computed tomography	Quantify renal sinus fat volume and validate reproducibility	Single APDRS correlates with RSF segmentation (*r* = 0.80); inter-reader ICC = 0.98; single-slice protocol ICC 0.93 (intra)/0.86 (inter)	Gold-standard for renal sinus fat quantification and validation of MR/US methods	**Level II b** (prospective controlled human studies)
Proton-Density Fat-Fraction (PDFF) Mapping	Quantify renal parenchymal fat fraction and assess its link to oxygenation	CV <5%; detects fat 0.4%–50%; PDFF correlates with BOLD *R*₂* (↑ fat = ↑ hypoxia); IDEAL-IQ: PDFF ↑ in T2DM vs. controls (*P* < 0.001), correlates negatively with eGFR (*r* = –0.437) and positively with creatinine (*r* = 0.421); AUC 0.857, repeatability *r* = 0.81	Early metabolic phenotyping in T2DM, obesity, and HTN; longitudinal therapy monitoring; adjunct biomarker in multiparametric MRI	**Level II** a (prospective cohort + intervention)
Imaging + Omics Integration	Combine quantitative imaging with proteomics/genomics for prediction	CKD273 proteomic + T₁/ASL imaging models under validation—aim to enhance individualized risk stratification	Future precision medicine integration	**Level V** (conceptual/expert opinion)

**Table legend:** This table summarizes quantitative diagnostic findings, comparative clinical applications, and corresponding levels of evidence for each renal imaging modality discussed in the review. Ultrasound-based methods (CEUS, RRI, SMI, and SWE/ARFI) are contrasted with MRI-based biomarkers (BOLD, ASL, IVIM-DWI, *T*₁ mapping, and multiparametric MRI) that evaluate renal perfusion, oxygenation, fibrosis, and microstructure across CKD, DKD, hypertensive nephropathy (HTN), and transplant contexts. Levels of evidence (LOE) are graded according to the Oxford Centre for Evidence-Based Medicine Levels of evidence were graded according to the *Oxford Centre for Evidence-Based Medicine (OCEBM, 2023)* criteria [76]. **Abbreviations:** CEUS, contrast-enhanced ultrasound; CKD, chronic kidney disease; RRI, renal (intrarenal) resistive index; eGFR, estimated glomerular filtration rate; ACE I, angiotensin-converting enzyme inhibitor; SMI, superb microvascular imaging; SWE, shear-wave elastography; ARFI, acoustic radiation force impulse elastography; DKD, diabetic kidney disease; HTN, hypertension/hypertensive nephropathy; BOLD MRI, blood-oxygen-level-dependent MRI; *R*₂*, transverse relaxation rate (inverse of *T*₂*); SGLT2i, sodium–glucose cotransporter-2 inhibitor; ASL MRI, arterial spin labeling MRI; RBF, renal blood flow; IVIM-DWI, intravoxel incoherent motion diffusion-weighted imaging; DTI, diffusion tensor imaging; FA, fractional anisotropy; ESKD, end-stage kidney disease; *T*₁ mapping, native *T*₁ relaxation-time mapping; AUC, area under the receiver-operating characteristic curve; mpMRI, multiparametric MRI; CT, computed tomography; APDRS, anteroposterior diameter of renal sinus; RSF, region-scalable fitting segmentation; ICC, intraclass correlation coefficient; CKD273, urinary proteomic classifier (273 peptides for CKD prognosis); and LOE, level of evidence.

## US-BASED IMAGING

### Contrast-enhanced ultrasound

CEUS is the imaging technique that uses intravenously injected microbubble contrast agents to observe the renal microvascular perfusion. It is used when there is need for detailed kidney perfusion assessment, such as in hypertensive patients or early CKD in order to detect microcirculatory deficits also in evaluating renal lesions and transplant perfusions. It has been used as valuable tool to characterize indeterminate renal masses and to distinguish cortical necrosis from infarction. In contrast to conventional Doppler US, CEUS is more sensitive to low-flow in small vessels, that allows to detect perfusion abnormalities in capillaries that Doppler cannot visualize. It can also reveal abnormalities well before changes in routine labs or creatinine occur. Thus, CEUS picks up renal microvascular and perfusion abnormalities well before overt kidney injury. For example, CEUS revealed ∼28% lower baseline cortical perfusion in patients with essential hypertension compared to normotensive controls (median 1476 vs. 2062 arbitrary units; *P* < 0.001), along with a blunted cortical flow reserve during stress—hypertensive kidneys increased perfusion index by only ∼34% vs. ∼56% in controls [18]. This CEUS-derived perfusion index thus represents an immediate bedside read-out of microvascular damage that cannot be captured by routine laboratory tests [12]. Instead of single cutoff values, CEUS provides semi-quantitative perfusion indices. In clinical practice, delayed cortical enhancement or reduced peak intensity suggest problem with microperfusion. For example, in dynamic CEUS, a prolonged time to peak or lower peak intensity in the cortex suggests fibrosis and hypoperfusion in patients with CKD. While there is no universal cut off value, low cortical perfusion intensity such as 1500 arbitrary units compared to more than 2000 in healthy tissue or a reduced perfusion response under stress are warning signs of microvascular damage. CEUS is a fast, reliable, and cost-effective imaging tool, which can remove the need for other more expensive imaging techniques such as computed tomography (CT) or MRI. It is a fast, reliable, and cost-effective imaging tool that avoids ionizing radiation and nephrotoxic contrast exposure, making it particularly suitable for patients with impaired kidney function. Across studies, CEUS consistently shows that patients with hypertension and early CKD have moderate to large reductions in kidney cortical blood flow, usually about 25%–30% lower than in healthy controls, along with a reduced ability to increase blood flow when needed [12, 18]. These findings are seen in both hypertensive and CKD populations, showing good biological consistency, although the exact numbers differ because CEUS provides semi-quantitative measurements. Delayed cortical enhancement, reduced peak intensity, and impaired stress responses repeatedly emerge as clinically meaningful indicators of microvascular dysfunction, despite the absence of universal cutoff values [12, 18].

### Doppler resistive index

The intrarenal resistive index (RRI) is the Doppler US measurement that shows the blood flow through the kidney small arteries. It is calculated using waveforms from segmental or interlobar arteries during a standard renal US. As RRI doesn’t depend on probe angle, it reliably indicates the degree of vascular resistance and the health of small renal vessels. Conventional Doppler US findings align with these results. Once the RRI exceeds ∼0.70—especially as it approaches 0.80—it predicts a faster decline in eGFR and a higher rate of cardiovascular events, even in hypertensive patients with well-controlled blood pressure [19]. In DKD, similarly elevated RRI values (≈0.71–0.72) accompany more advanced nephropathy and higher albuminuria [20]. More broadly, in CKD patients an RRI ≥0.65 signifies extensive interstitial fibrosis and arteriosclerosis and portends a high risk of rapid renal function decline [21] . Moreover, each 0.01 increment in RRI has been associated with a 4% increase in mortality hazard and a 6% increase in dialysis progression hazard over ∼6 years, independent of blood pressure control [22]. Importantly, interventions that improve intrarenal hemodynamics can lower RRI—24 months of ACE inhibition (lisinopril) reduced RRI from ∼0.61 to 0.56 alongside a three-fold drop in microalbuminuria, whereas calcium channel blockade (nifedipine) had no effect [23]. Conversely, an extremely elevated RRI (≥0.80) identifies patients at particularly high risk, correlating with faster creatinine rise and nearly doubled long-term mortality in non-proteinuric CKD [24]. Because RRI is measured during a routine Doppler US, without contrast or radiation, and shows consistent results when standardized, it shows cost effective biomarker of renal blood flow (RBF). That makes it valuable for routine evaluation of both native and transplanted kidneys. Across the hypertensive, diabetic, and CKD populations, elevated intrarenal RRI has been linked to worsening renal function and higher cardiovascular risk. Values above 0.7 are commonly associated with faster declines in eGFR, while values near or above 0.8 has been linked to a particularly high-risk group [19–22, 24]. Although RRI can be affected by overall blood pressure and vascular conditions, its good reproducibility and strong links to clinical outcomes make it one of the most practical ultrasound markers for everyday clinical use.

### Superb microvascular imaging

Superb microvascular imaging (SMI) is an advanced Doppler US technique that visualizes low velocity blood flow in very small blood vessels without using contrast. SMI is an emerging technology currently available in select US systems and its use is growing for difficult to diagnose cases. It is useful when fine-detail perfusion of the renal cortex is desired but there is no availability of contrast or MRI. In clinical practice, it can be used during standard US exam to assess cortical microvasculature in CKD, to evaluate the vascular patterns in tumor, enabling differentiation of malignant vs. benign lesions based on flow pattern, or to monitor the RBF changes after treatments. It is especially valuable detecting microvascular rarefaction in CKD patients noninvasively. SMI primarily provides qualitative and semi-qualitative data. Although there are no standardized numeric thresholds, SMI offers a visual threshold—if small vessels are barely detectable where they should be abundantly seen, then microcirculatory damage is likely present. In contrast to Doppler, SMI can directly visualize small-vessel blood flow that was previously below Doppler detection. Renal SMI resolves cortical vessels as small as ∼300–500 μm, revealing fine-scale perfusion heterogeneity that closely mirrors perfusion deficits seen on arterial spin labeling (ASL) MRI and can even show improvements with therapy [12]. This high-frame-rate technique adds granularity to noninvasive microvascular assessment, visualizing the small-vessel rarefaction that conventional Doppler could only infer [12]. In contrast to CEUS, SMI doesn’t require contrast injection, so it can be used in patients who can’t receive contrast. SMI is essentially a software feature on modern US machines, so once the equipment is available, the incremental cost of using SMI is negligible. By providing microvascular detail during a routine US, SMI can potentially reduce the need for more expensive tests. Across available studies, SMI provides clearer visualization of reduced microvascular blood flow in the kidney cortex by detecting low velocity flow which is below the sensitivity of conventional Doppler techniques. These findings are consistently seen across CKD populations, but interpretation is still mostly visual and semi-quantitative because there are no standardized numeric cutoffs. The differences mainly arise from technical factors, not real biological changes, so SMI works best as a supporting, not independent, biomarker [12].

### US elastography

US elastography techniques measure tissue stiffness as a surrogate for fibrosis and can be performed during a routine renal US. A 2023 meta-analysis of 1394 participants demonstrated that shear-wave elastography (SWE) can noninvasively distinguish biopsy-proven renal fibrosis, with area under the receiver operating characteristic curve (AUROC) values ∼0.87 for mild, 0.78 for moderate, and 0.86 for severe fibrosis [25]. Consistently, in 162 biopsy-verified CKD cases, every 10 kPa reduction in cortical stiffness corresponded to a 4.5-fold higher odds of moderate-to-severe fibrosis on histology [26]. In DKD, cortical SWE values around 19 kPa identified late-stage DKD with ∼93% sensitivity and ∼86% specificity [20]. Moreover, a lower stiffness cutoff (∼9.2 kPa) could differentiate early-stage DKD (CKD stages 1–3) from healthy controls (67% sensitivity, 82% specificity) [27]. Notably, beyond renal outcomes, a radiomics nomogram combining six SWE-derived features with clinical factors predicted incident cardiovascular disease in DKD with an AUC ≈ 0.89 [28]. Hypertensive nephropathy shows a similar fibrotic imprint: acoustic radiation force impulse (ARFI) elastography found significantly lower cortical shear-wave velocity in chronic hypertension (∼2.37 m/s) compared to healthy kidneys (∼2.96 m/s), correlating with higher albuminuria (*r* = –0.76) and lower eGFR (*r* = 0.78) [29]. ARFI not only differentiates hypertensive kidney disease from normal tissue, but also correlates with hypertension grade and kidney damage severity, supporting its use as an objective, operator-independent endpoint in reno-protective trials [29]. Elastography is attractive because it is noninvasive, repeatable, and operator–independent when standardized that can potentially reduce need for renal biopsy. Elastography introduces minimal extra cost as it is a standard software module on modern US platforms, while providing wide clinical uses in many diseases such as DKD, hypertensive nephrosclerosis, CKD of unclear etiology, and renal transplant surveillance. Across CKD, DKD, and hypertensive nephropathy, renal elastography has shown strong ability to detect kidney fibrosis, with AUROC values generally in the 0.78–0.87 range. While in early CKD, the overlap with normal kidney tissue makes fibrosis harder to distinguish, its performance tends to be more reliable in advanced disease. Although reported stiffness thresholds appear promising in practice, they still depend on the imaging platform, with heterogeneity driven by technical factors and perfusion effects [25–27, 29]

## MRI TECHNIQUES

### Blood-oxygen-level-dependent MRI

Blood-oxygen-level-dependent (BOLD) MRI assesses tissue oxygenation. In a prospective study of 112 CKD patients, elevated cortical *R*₂* (indicating low oxygenation) independently predicted a three-fold higher risk of progression to end-stage outcomes over ∼3 years [30] . An outer-cortex *R*₂* above the 90th percentile likewise conferred a ∼3× risk of renal replacement therapy or ≥30% creatinine increase in hypertensive CKD, even after adjustment for baseline eGFR, proteinuria, and other factors [30]. Advanced BOLD methods confirm true tissue hypoxia: in stage 3–4 CKD, uncorrected *R*₂* values no longer correlated with GFR, whereas iron oxide-corrected BOLD imaging clearly showed cortical and medullary hypoxia. Furthermore, acute changes in cortical oxygenation can be captured. In albuminuric diabetic patients, a single 50 mg dose of the SGLT2 inhibitor dapagliflozin lowered cortical *R*₂* by ∼9% within 6 h [31], reflecting improved oxygenation that would be undetectable by standard lab measures. Although BOLD MRI requires additional scanner time and technical expertise, it is a non-contrast technique that can be incorporated into multiparametric MRI protocols and provides valuable physiologic information for both risk assessment and treatment monitoring. While its thresholds are relative and vary with magnetic field strength, higher cortical *R*₂* values consistently correspond to greater disease risk. Across CKD and hypertensive populations, higher cortical *R*₂ values on BOLD MRI* are consistently associated with worse renal outcomes, including about a three-fold higher risk of disease progression [32, 33]. Absolute *R*₂* thresholds vary substantially due to differences in field strength, hematocrit, and correction methods, making relative comparisons and longitudinal changes more reliable than fixed cutoffs. These features support BOLD MRI as a sensitive functional biomarker rather than a static diagnostic measure.

### ASL MRI

ASL MRI provides quantitative, contrast-free maps of RBF. ASL studies consistently show reduced perfusion in CKD. Cortical RBF may be nearly halved by stage 3 CKD compared to healthy kidneys, and flow correlates with eGFR [4, 5]. For example, a threshold of ∼143 ml/min/100 g distinguished CKD patients from controls with an AUROC ∼0.98 [34]. ASL measurements decline progressively with advancing CKD stage and have a rank correlation ∼0.8 with eGFR [35]. Even in patients with preserved GFR, ASL can uncover hidden deficits: in biopsy-proven early CKD (eGFR ≥ 90), cortical ASL-RBF and IVIM-derived perfusion fraction *f* were significantly lower than normal; perfusion fraction achieved an AUC ∼0.92 for detecting early injury [26, 36]. In diabetes, ASL-based radiomics can detect subclinical disease: one radiomic signature differentiated normoalbuminuric ss from healthy controls with AUC 0.865, and a combined imaging–clinical model predicted progression to overt nephropathy with ∼74% accuracy (AUC 0.734) [37]. Because ASL is contrast–free, quantitative, and now standardized in research protocols, it is increasingly feasible to incorporate as a cost–effective biomarker within a single multiparametric MRI exam to phenotype ischemic patterns and monitor responses without gadolinium. ASL MRI consistently demonstrates a significant decrease in cortical kidney blood flow as CKD advances, with perfusion values frequently falling to almost half by stage 3 illness and exhibiting a strong correlation with eGFR. Perfusion values of about 143 ml/min/100 g consistently distinguish patients with CKD from healthy controls, and the method has a high diagnostic accuracy [34–36]. Rather than actual biological diversity, differences between research are primarily due to technical issues.

### Intravoxel incoherent motion–diffusion-weighted imaging

Diffusion MRI, including intravoxel incoherent motion (IVIM) and diffusion tensor imaging (DTI), assesses renal microstructure and microvascular flow. In a biopsy-phenotyped CKD cohort, a lower cortical IVIM perfusion fraction *f*—and, to a lesser extent, a reduced true diffusion coefficient *D*—were independently associated with progression to end-stage kidney disease over five years. Adding *f* and *D* to a model with baseline eGFR and fibrosis improved the prognostic AUC from 0.886 to 0.955, and each 1% drop in f corresponded to ∼21% higher ESKD risk [38]. Diffusion metrics also flag subtle diabetic kidney injury. In type 2 diabetes mellitus (T2DM), medullary diffusion parameters showed high diagnostic power for incipient nephropathy: the IVIM-derived true diffusion (*D*) achieved AUC ∼0.95 and DTI-based fractional anisotropy (FA) ∼0.91 for distinguishing early (microalbuminuric) DKD, and combining D with FA yielded near-perfect discrimination (AUC 0.99) [39]. While absolute thresholds vary by scanner and protocol, lower *f* and *D* and lower medullary FA signify worse microvascular/tubular integrity. Because diffusion sequences are fast, contrast–free, and easily appended to MRI, they provide a cost–efficient route to risk–stratify patients and identify fast progressors in targeted clinical pathways and trials. Lower IVIM perfusion fraction and diffusion measurements are consistently associated with worse outcomes and disease progression in biopsy-characterized CKD and diabetic patients. Although the precise cutoff levels vary based on the scanner and imaging methodology, the observed effects are clinically significant [38, 39]. Diffusion MRI is a helpful predictive supplement rather than a stand-alone diagnostic technique because the majority of the variability is technical rather than biological.

### Fibrosis imaging (*T*₁ mapping)

Mapping of native (non-contrast) *T*₁ relaxation times provides a surrogate of tissue fibrosis. In a cohort of 119 CKD patients (G1–G4) followed for up to 3 years, those with the highest cortical *T*₁ values had a three-fold higher risk of requiring renal replacement or having a ≥30% rise in creatinine; adding *T*₁ to a clinical model improved the event prediction AUC from 0.83 to 0.88 [40]. Similarly, *T*₁ mapping can detect fibrotic changes in DKD. A recent study using radiomics of cortical *T*₁ maps achieved an AUC of ∼0.93 for any fibrosis and ∼0.90 for >25% fibrosis, outperforming models based on Δ*T*₁ or eGFR alone. The same *T*₁-based radiomic signature detected biopsy-proven fibrosis with 82% sensitivity and 91% specificity (AUC ∼0.93) in a training cohort, and yielded AUC 0.94 with 100% sensitivity and 86% specificity on validation [41]. These findings support native *T*₁ mapping as a noninvasive imaging biomarker of renal fibrosis and prognosis. Although absolute cutoffs differ by field strength and sequence, a higher–than–expected cortical *T*₁ and/or a rising *T*₁ trend over time flag significant scarring. *T*₁ mapping is quick, reproducible, and contrast–free, making it practical to integrate into multiparametric exams for risk stratification and longitudinal monitoring. Across different stages of CKD, renal fibrosis and worse clinical outcomes, such as a higher risk of creatinine advancement or the necessity for renal replacement treatment, are consistently associated with higher cortical native *T*₁ levels. Direct comparison between studies is limited since the exact *T*₁ cutoff values vary depending on the scanner and imaging sequence. Therefore, changes over time and relative increases in cortical *T*₁ are more therapeutically valuable than constant thresholds.

### Proton-density fat-fraction mapping

Proton-density fat-fraction (PDFF) is a Dixon-based quantitative MRI metric that reports the fraction of fat protons relative to total (fat + water) protons and resolves cortical versus medullary steatosis with a coefficient of variation <5% [42, 43]. In T2DM, higher cortical PDFF parallels increases in BOLD R2*, consistent with hypoxia, linking lipid load to oxygenation changes [40]. Whole-kidney PDFF maps are acquired in ∼3–4 min, detect fat fractions from ∼0.4% to >50%, and are more accurate than CT for parenchymal fat quantification; PDFF can be incorporated alongside intravoxel incoherent motion–diffusion-weighted imaging (IVIM/DWI) and native T1/T2 mapping within a single multiparametric session [15, 44]. Clinically, PDFF is most useful in metabolic phenotypes (T2DM, obesity, hypertension) for early phenotyping—when albuminuria may precede a measurable change in GFR—and for longitudinal therapy monitoring within the same visit [15, 43, 44]. Among advanced implementations, iterative decomposition of water and fat with echo asymmetry and least-squares estimation—iron quantification (IDEAL-IQ) demonstrates significantly higher renal parenchymal PDFF in T2DM vs. controls (*P *< 0.001), with PDFF correlating negatively with eGFR (*r* = –0.437) and positively with creatinine (*r* = 0.421); repeatability is excellent (*r* = 0.81; mean difference ∼0.004%), and diagnostic performance is strong (AUC 0.857 for distinguishing T2DM from controls), supporting clinical applicability as an adjunct biomarker [45]. Reporting should include PDFF (%) separately for cortex and medulla and acquisition details (field strength/sequence); while universally accepted disease thresholds are not yet established and may be scanner-dependent, center-specific reference ranges, longitudinal trends, and concordance with complementary MRI biomarkers (e.g. BOLD R2*) are recommended for interpretation [43–45]. Renal PDFF is consistently associated with reduced kidney function and hypoxic symptoms in all metabolic types of kidney disease. Although measurements are repeatable, precise disease-specific cutoff values have not yet been established and still rely on the scanner in use. Therefore, rather than rigid diagnostic classification, PDFF appears to be more helpful for early illness characterization and long-term follow-up.

### Multiparametric MRI and other advances

Many of the above MRI techniques can be combined into a single multiparametric scanning session. Modern 3 T protocols (≈45 min) now integrate phase-contrast flow mapping, ASL perfusion, BOLD *R*₂* mapping, diffusion-weighted imaging, and quantitative *T*₁ mapping [32]. Using such a multiparametric approach, a study of 38 diabetic CKD patients and 20 controls found that reductions in RBF (by phase-contrast MRI) and cortical perfusion (by ASL) most clearly distinguished DKD from healthy kidneys [32]. Furthermore, in a 151-patient CKD cohort (including 29 DKD) followed for ∼3.8 years, a panel of four MRI biomarkers (perfusion, oxygenation, diffusion, and *T*₁) was evaluated: although all markers correlated with disease, only cortical BOLD *R*₂* remained an independent predictor of subsequent eGFR decline when all four were considered together [32]. These MRI sequences require no exogenous contrast and have shown robust repeatability across visits (within-subject coefficient of variation ∼4% for *T*₁ and diffusion parameters, ∼11% for perfusion) [46]. Accordingly, quantitative MRI is increasingly feasible for routine use to stratify “fast” vs. “slow” progressors and may ultimately serve as a noninvasive surrogate for biopsy in assessing renal fibrosis [32]. Additionally, when evaluation of renal vasculature is needed, non-contrast MR angiography techniques (e.g. time‐SLIP) can reliably detect renal artery stenoses with ∼90% accuracy vs. CT angiography [47]. While mpMRI requires scanner time and specialty expertise, bundling multiple contrast–free biomarkers into one visit can streamline care pathways and reduce repeated testing, supporting cost–effectiveness in specialized centers. By integrating measurements of perfusion, oxygenation, diffusion, and fibrosis into a single scan, multiparametric MRI adds prognostic value across several cohorts. Even after controlling for other variables, oxygenation-based metrics such cortical BOLD *R*₂* frequently continue to be independently predictive, despite the fact that many of these markers are linked to the severity of the disease. Current repeatability results indicate mpMRI as a potential method in specialized clinical contexts, despite technical complexity and restricted availability still being obstacles.

## CT

For quantification of the renal-sinus fat volume, non-contrast multidetector computed tomography remains the cornerstone imaging modality. A validation study of 98 out–patients in 2024 showed that a single anteroposterior diameter of the renal sinus (APDRS) correlates strongly with volumetric region-scalable fitting (RSF) segmentation (*r* = 0.80) and is highly reproducible [inter–reader intraclass correlation coefficient (ICC) = 0.98] [48]. Similarly, single-slice right-kidney protocols have shown to have high intra-reader ICC (0.93) and inter-reader ICC (0.86) reproducibility [49].

## MORE ON SPECIFIC CASE-USE IN KIDNEY DISEASE: FATTY KIDNEY AND KIDNEY TRANSPLANTATION

### Fatty kidney

**Figure 3:**
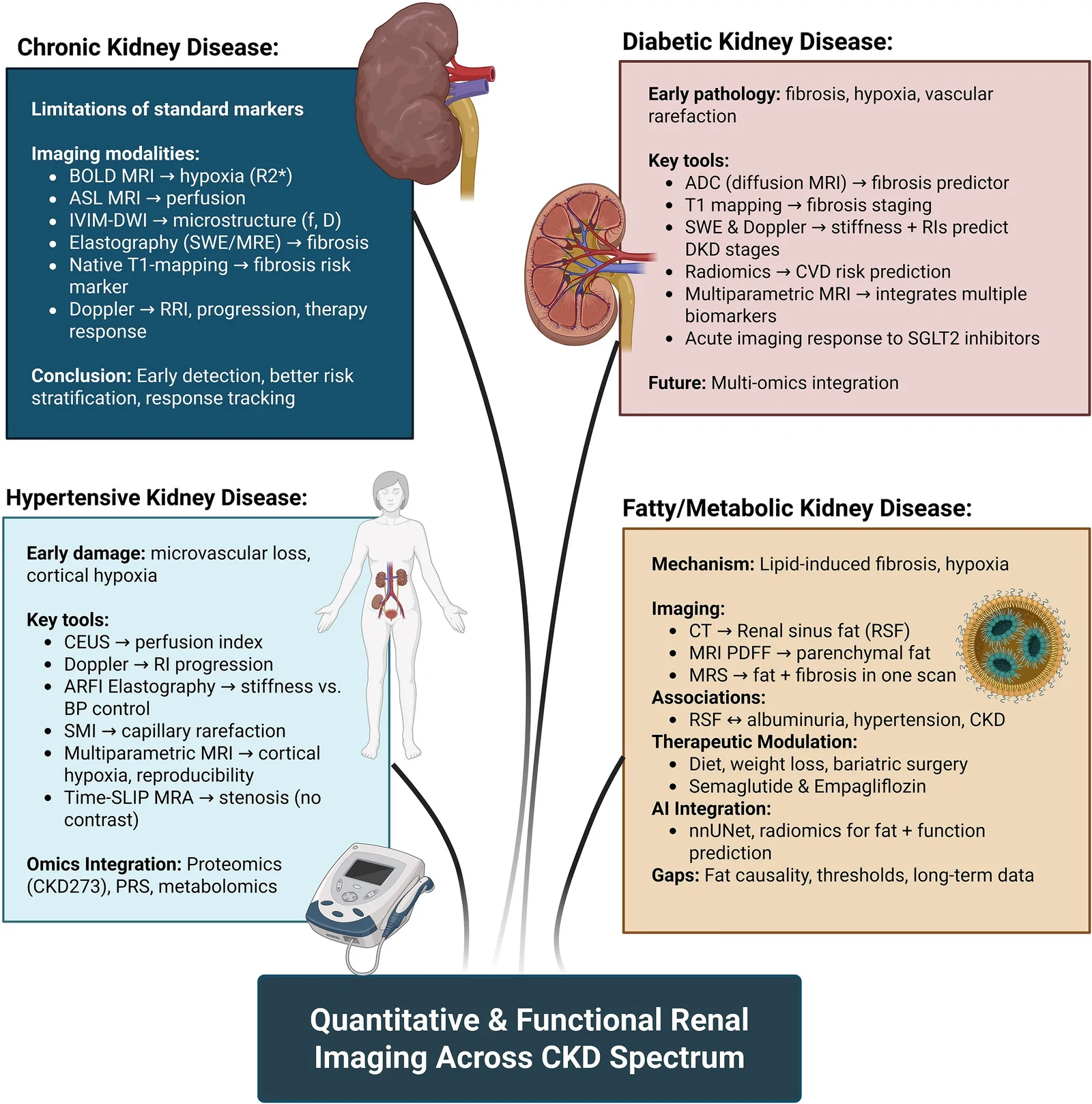
Closing the CKD diagnostic blind spot: early imaging detection slows disease progression. Conceptualized by the authors, informed by prior work (7). Figure legend: Integration of imaging into CKD diagnosis and management. Imaging enables detection of structural and functional renal changes before biochemical alterations become evident (e.g. eGFR decline or albuminuria). Early imaging-based identification allows earlier initiation of therapeutic interventions, which can slow or even halt disease progression. **Abbreviations:** CKD, chronic kidney disease; eGFR, estimated glomerular filtration rate; and UACR, urine albumin-to-creatinine ratio.

Renal adiposity represents a mechanistic extension of the early-injury paradigm, in which structural compression, microvascular dysfunction, and hypoxia precede measurable declines in eGFR. Imaging-based quantification of renal sinus and parenchymal fat therefore provides a direct link between metabolic injury, early risk stratification, and longitudinal prognostication (Fig. 3).

#### Metabolic and functional correlates

Community data from the Framingham Heart Study (*n* = 2923) showed that participants in the highest renal sinus fat decile had a 2.1–fold higher odds of hypertension and a 2.3–fold higher odds of CKD, after adjusting for visceral adipose tissue [50]. MRI studies further these findings: among 105 adults with T2DM, each 1% increase in cortical PDFF associated with a 12% higher odds of prevalent CKD after adjustment for body mass index and glycemic control [43]. Greater RSF has also been linked to albuminuria, hypertension and activation of the RAAS in primary aldosteronism, illustrating systemic-renal cross-talk [50–52].

#### Prognostic evidence

Prospective evidence, although still limited, indicates that renal adiposity is not only a passive marker of systemic obesity. In an 18–month randomized dietary trial (*n* = 278), higher baseline renal sinus fat predicted lower eGFR and greater micro–albuminuria, while overall renal sinus fat fell by 9% irrespective of diet [53]. Although long–term prospective data remains sparse, these observations suggest that renal adiposity is more than a passive marker of systemic obesity.

#### Therapeutic modulation

The impact of therapeutic modulation by lifestyle, surgery and medication on renal sinus fat has been assessed. *Lifestyle*. Weight-loss interventions consistently reduce renal sinus fat; the 18-month randomized dietary trial demonstrated a significant reduction in renal sinus fat (−9%; *P* < 0.05 vs. baseline) [53]. *Bariatric surgery*. Whole-body MRI in 74 severely obese patients revealed a ∼30 % decrease in renal sinus fat six months after sleeve gastrectomy, and the magnitude of renal sinus fat regression independently predicted hypertension remission [54]. *Pharmacotherapy*. In a 32-week randomized study of high-cardiovascular-risk T2DM, both semaglutide and empagliflozin reduced cortical apparent diffusion coefficient (ADC) and total kidney volume on diffusion-weighted MRI—changes interpreted as early micro-structural improvement before eGFR change [55]. These data support the inclusion of renal-fat metrics as adjunct end-points in intervention trials alongside albuminuria, C-reactive protein, and GFR slope.

#### AI and multimodal integration

Manual segmentation is a practical bottleneck. A 2024 European Radiology Experimental study reported that a 3-D nnU-Net achieved Dice scores ≥0.94 for renal segmentation on contrast- and non-contrast CT, enabling automated quantification of renal and perirenal fat within seconds [56]. Beyond segmentation, radiomic models that fuse non-contrast CT features with machine-learning algorithms now estimate split renal function with an AUC of 0.90, highlighting the potential of imaging-based phenotyping to complement eGFR [57].

#### Knowledge gaps and future directions in fatty kidney imaging

Despite growing evidence linking renal adiposity to kidney dysfunction, key uncertainties remain. These include whether renal-fat accumulation is causal or merely a risk marker, scanner-specific thresholds for defining “pathological” fat, and whether reductions in renal fat independently predict hard renal outcomes. Long-term, multicenter outcome studies are needed before renal-fat imaging can be fully integrated into clinical practice.

### Imaging of allograft kidney in kidney transplant recipients

In kidney transplant recipients, Doppler US remains the cornerstone of routine graft surveillance due to its non-invasiveness, availability, and ability to assess vascular integrity and perfusion. Standard protocols typically involve Doppler US within the first week post-transplant to evaluate early complications such as renal artery thrombosis, urinary leaks, and perinephric fluid collections [58]. Subsequent imaging may be performed at 1, 3, 6, and 12 months, although this varies by center and is often symptom-driven beyond the first year, and if there is a need to evaluate for complications [58].

In KT, imaging similarly enables detection of subclinical injury trajectories—such as microvascular dysfunction and progressive fibrosis—long before functional decline is apparent, positioning graft imaging as a natural extension of early-injury-based risk prediction into the post-transplant setting (Fig. 3).

The most critical US parameters for assessing transplant kidney function are RI for vascular resistance, echogenicity and size for parenchymal integrity, perfusion for vascular patency, and evaluation for hydronephrosis or collections indicating mechanical or surgical complications. RI is commonly measured, with elevated values (>0.8) associated with rejection, obstruction, or acute tubular necrosis. In cases of graft dysfunction or delayed graft function, adjunctive imaging modalities such as nuclear renography (e.g. Mercaptoacetyltriglycine scans), CT, or MRI/MRA might be utilized selectively. While biopsy remains the gold standard for diagnosing rejection or chronic changes, non-invasive techniques such as SWE, MRE, and photoacoustic imaging (PAI) are under investigation for detecting fibrosis, offering potential future tools for longitudinal graft assessment. PAI combines US and laser technology to detect acoustic signals from tissues, allowing for the quantification of collagen content and assessment of renal fibrosis.

As a key driver of chronic allograft dysfunction, renal allograft fibrosis typically evolves subclinically and is shown to predict long-term graft loss [59]. Renal allograft fibrosis is the progressive accumulation of extracellular matrix in the transplanted kidney, reflecting chronic structural injury from immune-mediated damage (e.g. chronic antibody-mediated rejection), ischemia-reperfusion, calcineurin inhibitor toxicity, and recurrent or de novo disease [59]. It manifests histologically as interstitial fibrosis and tubular atrophy, often with glomerulosclerosis and vascular remodeling [60].

Early detection of renal allograft fibrosis is essential, as significant histologic injury can be present even when serum creatinine and eGFR appear normal, and timely identification enables earlier intervention to prevent irreversible damage and prolong graft survival [60].

Fibrosis cannot be reliably detected with conventional US, and early fibrotic changes might typically go unnoticed. Diagnosis traditionally depends on biopsy, which is limited by sampling bias and risk [59]; emerging advanced or quantitative noninvasive modalities such as renal H-scan aim to quantify whole-organ fibrotic burden more accurately and reproducibly.

Registry-based analyses reinforce the prognostic value of serial RI assessment. In a large retrospective cohort (*n* = 1685) from France, trends in RI between 1 and 3 months post-transplant best predicted long-term risk of death with a functioning graft (DWFG); values ≥0.70 at both time points conferred nearly fourfold higher hazard (HR ∼3.77) relative to low RI at both times [61]. Another multicenter study (*n* ≈ 425) demonstrated that a ≥10% increase in RI between 4- and 12-months post-transplant independently predicted death-censored graft loss (HR ∼6.2; AUC ∼0.75) [62]. These findings underline the relevance of trajectory over absolute measurements and support structured Doppler surveillance in the first post-transplant year, even in the absence of symptoms. These findings suggest that structured longitudinal Doppler surveillance may support future risk-stratified screening strategies in large transplant populations.

## INTEGRATION OF AI AND RADIOMICS IN GENERAL NEPHROLOGY AND KIDNEY TRANSPLANTATION

AI and machine learning are increasingly applied across general nephrology, not only in KT. In non-transplant populations, AI-driven analysis of US and multiparametric MRI enables automated segmentation, quantitative assessment of perfusion, fibrosis, oxygenation, and tissue heterogeneity, and early risk stratification in CKD, DKD, and hypertensive nephrosclerosis. These approaches aim to identify subclinical injury patterns, predict disease progression, and support individualized therapeutic monitoring before irreversible functional decline occurs [13, 41].

In this context, deep-learning algorithms trained on multiparametric MRI and US datasets can automate segmentation, quantify perfusion, and estimate fibrosis. Deep-learning algorithms trained on multiparametric MRI and ultrasound datasets can automate segmentation, quantify perfusion, and estimate fibrosis. For example, the review by Zhao *et al*. summarizes multiple AI/machine learning imaging works in CKD [13]. Native *T*₁ mapping-based radiomics has been shown to differentiate renal fibrosis and functional decline with AUCs >0.9 in CKD populations.

### AI in diabetic and hypertensive kidney disease

Machine-learning frameworks combining imaging-derived metrics (e.g. elastography stiffness, ASL perfusion) with clinical variables have shown potential for detecting diabetic kidney injury. The study by Ma *et al*. used ASL-based radiomics and ML models to detect early diabetic renal damage [37]. In hypertensive nephrosclerosis, ARFI elastography has demonstrated increased renal stiffness correlating with fibrosis and other parameters, laying the groundwork for radiomics/ML in this domain [29]. Collectively, these studies demonstrate that AI-assisted imaging in general nephrology can move beyond descriptive assessment toward quantitative phenotyping, enabling earlier detection of fibrosis, perfusion deficits, and progression risk in CKD populations independent of transplantation.

Building on these advances in general nephrology, AI applications have been particularly active in kidney transplantation, where longitudinal imaging datasets and defined clinical endpoints facilitate model development.

### Machine learning and radiomics for US-based graft function prediction

The application of radiomics and machine learning to US imaging is a rapidly developing field in transplant nephrology. A 2022 study from Soochow University used a cohort of 233 kidney transplant recipients to develop machine learning models incorporating radiomic features extracted from gray-scale US images. These models achieved AUC values between 0.788 and 0.839 for differentiating between normal and impaired graft function, highlighting the potential of radiomics to enhance functional prediction beyond conventional imaging interpretation [63].

Building on this, a 2023 scoping review published in Transplantation cataloged 16 studies exploring radiomics and AI in kidney transplantation. These studies predominantly focused on predicting allograft rejection, fibrosis progression, or functional decline using a combination of imaging-derived features and clinical parameters. The review emphasized that while radiomics offers promising non-invasive biomarkers, the lack of standardization and small sample sizes remain major barriers to clinical implementation [64]. Nevertheless, these early efforts suggest that AI-powered radiomics could complement or even partially replace invasive biopsy in longitudinal monitoring of graft health.

### Radiomics in multiparametric MRI for graft survival prediction

Recent advances have also extended radiomics and machine learning applications to multiparametric MRI in renal transplantation. A 2025 study utilized dynamic contrast-enhanced MRI with deep-learning algorithms to predict long-term graft survival in transplant recipients. This approach involved extracting high-dimensional radiomic features that quantified microvascular perfusion and parenchymal heterogeneity. When combined with patient clinical profiles, these models showed high accuracy in predicting 1-year graft outcomes, suggesting a potential role for MRI-based radiomics as a prognostic tool in kidney transplantation [65].

Though promising, these techniques are still in early developmental stages, and validation in larger, multicenter prospective trials is necessary. Importantly, this integration of imaging data with machine learning bridges quantitative imaging science with precision nephrology and could reshape future approaches to allograft risk stratification.

### AI–augmented Doppler and functional US

AI is under investigation to being applied to enhance interpretation of Doppler and perfusion US in transplant imaging. A 2024 deep-learning fusion model for CKD (though not exclusively transplant recipients) improved detection of early fibrosis using multimodal US features, suggesting immediate translational potential in allograft care. Additionally, machine learning has been applied to US-derived deep learning workflows, automatically analyzing Doppler waveforms and microbubble dynamics in CEUS to quantify microvascular flow, though most systems are currently in proof-of-concept or diagnostic development stages. Given the increasing availability of Doppler image datasets, convolutional neural network-based tools targeting subclinical perfusion deficits or automated RI measurements represent a promising future direction for diagnostic augmentation.

## FUTURE DIRECTIONS AND RESEARCH GAPS

The future of kidney imaging lies in its integration with genetics, clinical biomarkers, AI, and structural and functional data to deliver precision medicine. Emerging techniques such as multiparametric MRI [66, 67], US-based radiomics [13], and H-scan ultrasonography represent promising alternatives to biopsy and conventional laboratory tests. However, several unresolved challenges must be addressed before these methods can be widely implemented in clinical nephrology (Table 2).

**Table 2:** Quantitative kidney imaging across disease phenotypes: read-outs, clinical utility, limitations, and research gaps.

Disease	Imaging and quantitative read-outs	Utility and incremental value	Limitations	Research gaps and recommendations
CKD	ASL → RBF; BOLD → *R*_2_*; DWI/IVIM → ADC, *f, D*; native *T*_1_ mapping; Doppler → RRI; SWE/MRE → stiffness	BOLD R2* (↓ oxygenation) predicts faster eGFR decline, adverse outcomes [30]. ASL-RBF falls stepwise with CKD stage, discriminates CKD vs. controls [34]. IVIM (*f, D*) and native T1 associate with fibrosis/progression beyond routine markers [40, 77].	BOLD influenced by hemodynamics/blood volume unless corrected; between-study heterogeneity [33]. US measures (Doppler/SWE) are operator-dependent; inter-vendor variability for MR sequences [19].	Multicenter harmonization of ASL/IVIM/T1/BOLD protocols & phantoms; embed ΔR2*, ΔRBF, ΔT1 as prespecified endpoints; integrate imaging with omics/ML for calibrated risk prediction [78].
DKD	ASL/PC-MRI → RBF; BOLD → R2*; DWI → cortical ADC; native T1 mapping; Doppler → RRI; SWE → cortical stiffness; multiparametric non-contrast MRI	Cortical ADC stratifies DKD stage and predicts outcomes beyond eGFR/UACR [79]. SGLT2 inhibitors acutely increase cortical oxygenation on BOLD-MRI (early pharmacodynamic read-out) [80]. Multiparametric MRI feasible for DKD phenotyping without gadolinium [81].	Many DKD studies are single-center; cross-scanner cut-offs not harmonized; US indices vary with technique [81].	Validate reproducible ADC/T1 thresholds across vendors; use multiparametric MRI as response markers in DKD trials; fuse imaging with urinary biomarkers (TNFR-1/KIM-1) and polygenic scores in ML models [78].
HKD	ASL → RBF; IVIM → *f, D*; T1 mapping; BOLD → R2*; Doppler → RRI; SWE → cortical stiffness; CEUS → perfusion index/flow reserve; time-SLIP MRA → RAS grading (non-contrast)	CEUS shows reduced cortical perfusion and decreased renal flow reserve in hypertension [18]. RRI ≥0.70 associates with higher mortality and CKD progression risk [82]. Time-SLIP non-contrast MRA accurately detects/grades renal artery stenosis (multicenter) [47].	CEUS/US are operator-dependent; BOLD requires hemodynamic context; limited biopsy-linked validation specific to hypertension [19, 33]	Standardise CEUS metrics and test imaging-guided therapy titration (e.g. RAAS/diuretics); expand non-contrast MRA validation in CKD cohorts [83, 84].
Fatty kidney/metabolic renal phenotype	Dixon MRI → PDFF; MRS → intrarenal lipid content; BOLD → R2*; CT → RSF (volumetric/APDRS); automated CT segmentation (DL)	Highest RSF decile linked to higher odds of hypertension and CKD independent of visceral fat [50]. APDRS correlates with volumetric RSF and shows excellent reproducibility [48]. Semaglutide/empagliflozin: early MRI signals (↓cortical ADC, ↓TKV) over 32 weeks [55].	No harmonised PDFF/RSF pathological cut-offs; vendor/sequence effects; CT radiation; causality unresolved [43].	Define PDFF/RSF thresholds across scanners/ethnicities; validate automated segmentation/radiomics across centres; test whether ΔPDFF/ΔRSF independently predict hard renal outcomes [48, 55].
Kidney allograft (transplant)	Multiparametric MRI → DWI/ASL/BOLD/T1/T2; Doppler → RRI trajectories; CEUS → cortical perfusion; SWE → cortical stiffness; MRE → whole-kidney stiffness; H-scan → fibrosis index; PAI (during NMP) → cortical sO₂	RI early/serial predicts delayed graft function and adverse outcomes [85]. SWE shows moderate correlation with Banff fibrosis (systematic review/meta-analysis) [86]. H-scan (first-in-human) correlates with 12-mo eGFR; PAI during NMP discriminates functional vs. non-functional grafts by cortical oxygenation [87, 88].	Inconsistent imaging–histology concordance (esp. MRE); heterogeneity in SWE thresholds; clinical-decision cut-offs lacking; limited multi-center standardization [86, 89].	Prospective imaging–biopsy correlation with standardized protocols; evaluate ΔT1/Δperfusion/ΔSWE as surrogate endpoints; integrate AI/radiomics into transplant registries; expand CEUS/PAI/H-scan validation across centres [90].

**Abbreviations:** ASL, arterial spin labeling; BOLD, blood-oxygen-level-dependent MRI (R2* reflects tissue oxygenation); CEUS, contrast-enhanced ultrasound; CKD, chronic kidney disease; DKD, diabetic kidney disease; DL, deep learning; DWI/IVIM, diffusion-weighted/intravoxel incoherent motion MRI (ADC = apparent diffusion coefficient; f–D = IVIM perfusion/diffusion components); eGFR, estimated glomerular filtration rate; H-scan, quantitative ultrasound fibrosis index; HKD, hypertensive kidney disease; MRA, magnetic resonance angiography; MRE, magnetic resonance elastography; MRS, magnetic resonance spectroscopy; NMP, normothermic machine perfusion; PAI, photoacoustic imaging; PDFF, proton-density fat fraction; RAS, renal-artery stenosis; RBF, renal blood flow; RRI, renal resistive index [intrarenal Doppler; (PSV–EDV)/PSV]; RSF, renal-sinus fat; sO₂, oxygen saturation; SWE, shear-wave elastography; TKV, total kidney volume; UACR, urinary albumin-to-creatinine ratio; and US, ultrasound.

### Limitations and cause of variation

Several obstacles today impede the broad clinical use of quantitative renal imaging biomarkers. Technical restrictions include differences between scanners and suppliers, differences in acquisition methodologies and post-processing pipelines, and the lack of universally recognized thresholds for modalities including as SWE, BOLD MRI, ASL, and native T1 mapping. Biological variables such as hydration state, blood pressure, anemia, and concurrent cardiovascular sickness all have an impact on imaging results and complicate cross-study comparisons. Lastly, implementation barriers include cost, scanner availability, a lack of nephrology-focused imaging expertise, and the lack of support from guidelines hinder routine clinical use. In addition, while many studies report promising associations with fibrosis, progression, or outcomes, negative or equivocal findings and modest effect sizes have also been described, underscoring the need for larger, multicenter, and longitudinal validation before imaging biomarkers can be widely used as surrogate endpoints [3, 67, 68].

Validation and generalizability. A key limitation is the lack of longitudinal, multicenter validation studies, including repeated T1 mapping or ASL measurements within established CKD cohorts. Most imaging biomarkers have been validated only in small, often cross-sectional datasets, which undermines their prognostic value and generalizability [67, 68]. For instance, although T1 mapping and DWI correlate with fibrosis and declining kidney function [68, 69], their ability to reliably predict therapeutic response in DKD or across CKD stages remains uncertain.

### Clinical trial design

Another gap lies in the limited use of imaging as primary or secondary endpoints in clinical trials. To enable imaging-guided staging, stratification, and therapeutic monitoring, trial designs must be reconfigured. While regulatory bodies such as the United States Food and Drug Administration and European Medicines Agency have expressed openness toward imaging-based surrogate endpoints, standardized acquisition protocols and analytic reproducibility are prerequisites [3, 70].

### Multi-omics and AI integration

Future research should also advance the integration of imaging with transcriptomic, proteomic, metabolomic, and genetic datasets. This multimodal approach could generate high-resolution patient phenotypes, redefine clinical staging, and individualize therapy. Urinary proteomic classifiers (e.g. CKD273), metabolomic signatures, and polygenic risk scores have shown promise in predicting accelerated eGFR loss and cardiovascular risk beyond standard variables. Combining these molecular readouts with quantitative imaging markers—such as cortical T1, ASL perfusion, or elastography-based stiffness—may substantially refine risk stratification in kidney disease, although prospective validation is still required [71, 72]. The integration of such high-dimensional data through AI and machine learning offers further opportunities, though barriers remain, including real-time incorporation into electronic health records, external validation, and model interpretability [71, 73].

A notable shortcoming exists in the utilization of imaging for monitoring treatment interventions. While SGLT2 inhibitors, MRAs, and anti-fibrotic agents show promise in slowing CKD progression, there is less data regarding the use of imaging as an objective, non-invasive measure of therapy response. Future research may enhance treatment decision-making by evaluating how changes in imaging measures (e.g. cortical T1, renal perfusion) relate to therapy outcomes [69, 74].

Ultimately, implementation obstacles—such as financial constraints, accessibility issues, insufficient nephrology-focused training in imaging interpretation, and the lack of inclusion in guidelines—further impede practical application. National initiatives, such as the UK’s NURTuRE imaging substudy [75], and European consortia like PARENCHIMA (3) are commencing efforts to overcome these obstacles via standardization and multicenter validation.

In conclusion, although kidney imaging is set to revolutionize prognostication and treatment oversight in nephrology, achieving this potential necessitates focused research in five critical domains: (i) longitudinal and multicenter validation [67, 70], (ii) imaging-informed clinical trials [69], (iii) multi-omics integration and AI-driven modeling [13, 73], (iv) therapeutic monitoring endpoints [69, 74], and (v) implementation science to enhance guideline incorporation and equitable access [3, 75].

Integrating these high-dimensional imaging maps with emerging fluid biomarkers such as urinary tumor necrosis factor receptor 1 (TNFR-1) and kidney injury molecule 1 (KIM-1), as well as polygenic risk scores—and interrogating the ensemble through machine learning—hints at a future where DKD is staged not by creatinine and albuminuria but by truly multidimensional, actionable endotypes. Prospective multicenter trials are now needed to validate these imaging biomarkers as surrogate endpoints and to assess their cost-effectiveness in precision nephrology.

### Clinical translation and practical implications

From a clinical perspective, several renal imaging tools discussed in this review are already suitable for implementation in routine practice, while others remain investigational. Currently implementable approaches include Doppler-derived renal resistive index and ultrasound elastography, which can be used as supportive markers of intrarenal vascular resistance and fibrosis in selected contexts such as CKD progression assessment, hypertensive nephrosclerosis, and kidney transplant surveillance, particularly when interpreted longitudinally rather than as isolated measurements [19, 21, 25, 29]. Promising but still research-grade modalities include multiparametric MRI techniques—such as native T1 mapping, ASL-derived perfusion, and diffusion-based metrics—and their radiomics extensions, which have demonstrated strong associations with fibrosis, hypoxia, and outcomes but currently lack standardized thresholds, multicenter validation, and guideline endorsement [3, 32, 67]. Finally, primarily experimental techniques, including photoacoustic oxygen mapping, H-scan ultrasound-derived fibrosis indices, and advanced AI-driven multimodal models, remain confined to specialized research settings and early-phase clinical studies [12, 64, 73]. Explicitly distinguishing these tiers may help clinicians integrate available imaging tools into current care pathways while anticipating future developments as evidence matures.

**Figure 4:**
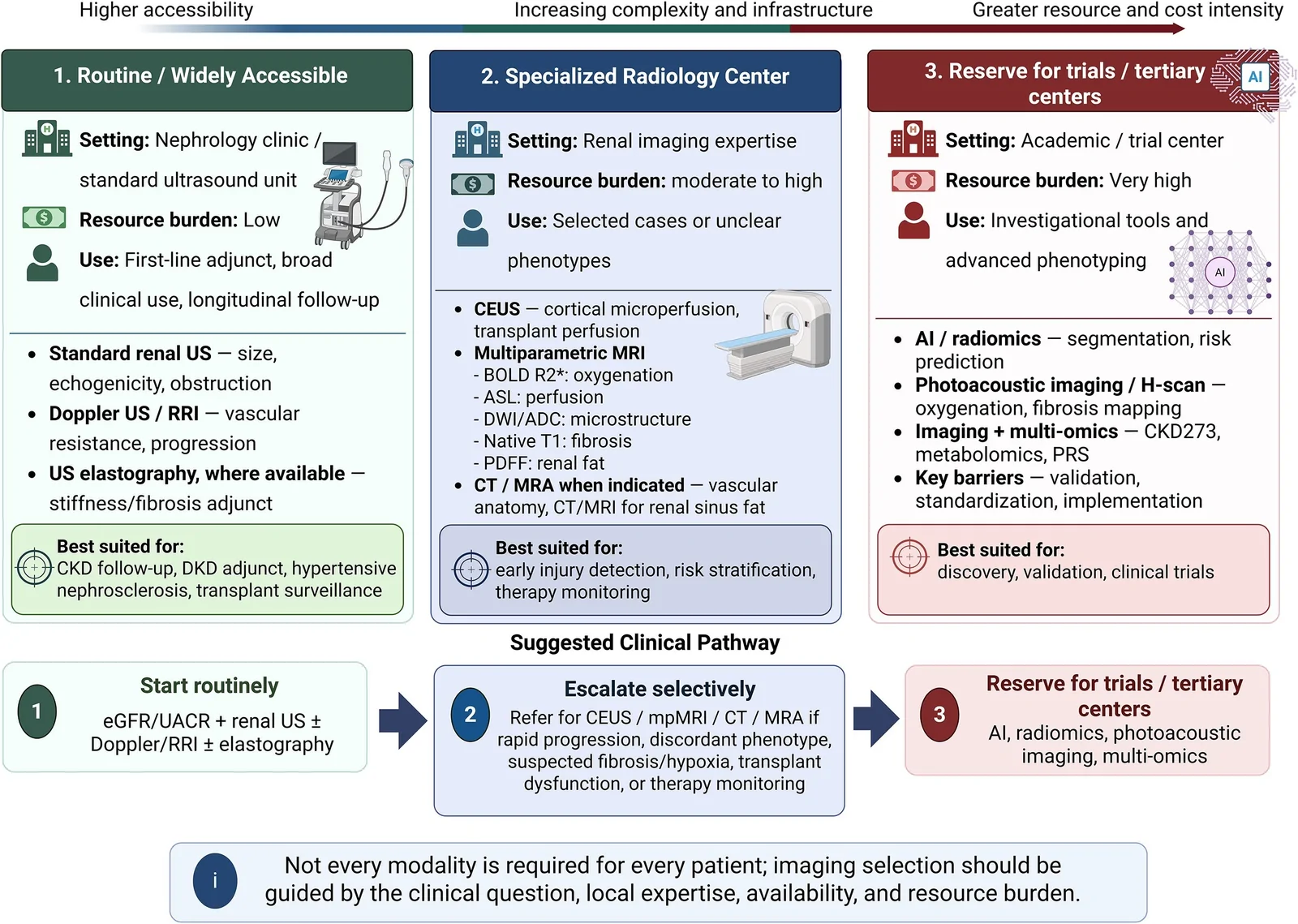
A stepwise imaging pathway for CKD based on clinical complexity and resource availability. Figure legend: Proposed framework for integrating imaging into CKD assessment according to clinical indication, expertise, and resource requirements. Routine evaluation begins with renal ultrasonography, Doppler ultrasound/RRI, and elastography where available. Patients with uncertain findings or higher-risk disease may be referred for CEUS, mpMRI, or CT/MRA. Emerging techniques, including AI, radiomics, PAI, and imaging–multi-omics, are primarily intended for research and tertiary centers. Imaging should be selected according to the clinical question, local expertise, and availability. **Abbreviations:** AI, artificial intelligence; ASL, arterial spin labeling; BOLD, blood oxygen level-dependent; CEUS, contrast-enhanced ultrasound; CKD, chronic kidney disease; CT, computed tomography; DWI, diffusion-weighted imaging; ADC, apparent diffusion coefficient; mpMRI, multiparametric magnetic resonance imaging; MRA, magnetic resonance angiography; PDFF, proton density fat fraction; PRS, polygenic risk score; RRI, renal resistive index; UACR, urine albumin-to-creatinine ratio; and US, ultrasound.

As summarized in Fig. 4, a pragmatic, tiered imaging pathway is proposed in which widely available ultrasound-based techniques serve as first-line tools for routine CKD assessment, while advanced modalities such as CEUS, multiparametric MRI, and CT/MRA are reserved for selected clinical indications. Emerging technologies, including AI-assisted imaging, radiomics, PAI, and imaging–multi-omics, are currently best suited for research and tertiary care settings, reflecting the need to balance diagnostic value with resource availability and clinical complexity.

## ONGOING TRIALS

A variety of current clinical trials (Table 3) are investigating new renal imaging biomarkers in diverse populations affected by CKD, DKD, and kidney transplants. Multiple trials seek to establish a correlation between multiparametric MRI parameters (such as T1/T2 mapping, ASL, and ADC) and histopathological markers, including fibrosis, inflammation, and age-related decline. Some researchers utilize hyperpolarized^13C MRI to detect metabolic changes, including glycolysis and lactate accumulation, thereby providing insights into early disease phenotypes. A segment of research investigates repeatability through comparisons of scans acquired at brief intervals, while other studies evaluate imaging performance relative to gold-standard benchmarks, such as biopsy or renal perfusion assessed via CEUS. Using MRI and positron emission tomography (PET), a multicenter research study tracks DKD imaging indication. This implies that the prediction of imaging outcomes is being evaluated. There is a growing interest in the application of advanced imaging to predict the progression of a disease, ensure that the appropriate medication is administered to the appropriate individual, and enhance the accuracy of non-invasive kidney tests.

**Table 3:** Summary of clinical trials investigating advanced kidney imaging modalities in CKD and transplantation.

Clinical trial number	Study title	Status	Study type	Patient population	Imaging modality & exposure	Primary imaging or clinical outcome	Secondary outcomes/biomarkers
NCT06202235	Multifrequency Renal MR Elastography in Evaluation of CKDs: Can Shear Stiffness Evaluate Renal Fibrosis in GFR-normal Patients?	Recruiting (28/04/2023)	Observational	Target sample size: 100 Adults aged 18–65 years with CKD stages 1–5, undergoing renal MRI and biopsy	Multifrequency magnetic resonance elastography (MRE)	MRE accuracy in identifying renal fibrosis in CKD [Time Frame: 12 months]	
NCT06210555	Multiparametric MRI in a Prospective Cohort of Living Kidney Donors, Recipients, and Healthy Controls: Correlations with Markers of Renal Function, Fibrosis, and Ageing	Recruiting (29/10/2024)	Observational	Target sample size: 96 Living kidney donors, transplant recipients, and healthy controls ages 18–80	Multiparametric MRI: T1/T2 mapping, ADC, ASL	Correlation between MRI parameters and fibrosis from renal biopsy [Time frame: up to 24 months]	GFR, biomarkers, aging-related decline, predictive modeling of allograft fibrosis
CTIS2024-512491-35–00	CKD—imaging the metabolic derangements with ultra-sensitive MRI	Recruiting (01/04/2024)	Interventional (Therapeutic exploratory, Phase II)	Target sample size: 30 Adults aged 18–85 years old; 8 healthy, 12 CKD (eGFR > 60), 10 ADPKD patients.	Multiparametric MRI using hyperpolarized [1–13C] pyruvate	MRI metabolic signature of the kidney: glycolysis shift, lactate/alanine production, fibrosis/inflammation correlation	Kidney function (blood/urine), expired CO₂ measures
NCT03716401	Prognostic Imaging Biomarkers for DKD	Recruiting (01/09/2018)	Observational	Target sample size: 500 Adults aged 18–80 years old with Type 2 diabetes and eGFR ≥30 ml/min/1.73 m^2^	MRI, US, PET, microvascular assessment, and renal biopsy	Cross-sectional imaging biomarkers and renal biopsy correlation [Time Frame: 2 years]	Longitudinal imaging/biopsy marker assessment [Time Frame: 4 years]
NCT06159439	Validation of Contrast Enhanced Ultrasound (CEUS) for the Assessment of Renal Perfusion Using Renal MRI in CKD	Not yet recruiting (02/01/2024)	Interventional, Single Group Assignment	Target sample size: 10 Adults with CKD stages 3b or 4 (non-ADPKD), eligible for MRI and CEUS	CEUS and ASL-MRI scans	Correlation between ASL-MRI measures of cortical perfusion and CEUS measures of renal microvascular blood flow.	CEUS vs MRA perfusion metrics, PC-MRI flow, CEUS repeatability
NCT03578523	Using MRI ASL Techniques for Quantitative Measurement of Renal Hemodynamics and Structural Parameters in Acute Kidney Injury and CKD	Unknown status (October 2015)	Observational	Target sample size: 50 Adults aged >18 and <95 years old with CKD stages 3–4	3 Tesla multiparametric MRI (diffusion weighted imaging, T1, volume), iohexol clearance, renal biopsy	Differences in DWI, volume, and T1 MRI parameters between healthy, CKD, and AKI patients [Time frame: 3 years]	Correlation with eGFR, creatinine, renal biopsy, blood/urine sampling
				(eGFR 20–60) and AKI stage 2–3. Excludes transplant patients.			
NCT03705091	Non-Contrast MRI to Measure Renal Transplant Perfusion and Fibrosis—Association with Function and Prognosis	Unknown status (11/07/2017)	Observational	Target sample size: 20 Adults aged 18–80 years old with recent or pending kidney transplantation	Magnetic resonance imaging	Change in arterial spin labeling (ASL) [Time Frame: Measured at 2, 6, and 12 months post-transplant]	Change in ADC [Time Frame: Measured at 2, 6, and 12 months post-transplant]
NCT04508049	Quantitative Magnetization Transfer MRI for Evaluation of Renal Fibrosis	Active, not recruiting (01/10/2020)	Interventional, Single Group Assignment	Target sample size: 24 Adults aged 40–80 years old with renovascular hypertension, eGFR >30; no transplant	Quantitative Magnetization Transfer MRI (qMT-MRI)	Fibrosis in stenotic and contralateral kidneys using qMT-MRI [Time Frame: Baseline]	Comparison of fibrosis to kidney function and injury markers
NCT05229263	Exploratory Multicenter Clinical Study to Assess Repeatibility, Reproducibility, Acceptability, and Clinical Validity of Multiparametric Renal Magnetic Resonance Imaging	Active, not recruiting (25/11/2022)	Interventional, Non-Randomized, Parallel Assignment	Target sample size: 140 Healthy adults and CKD stage 2–3 patients, ages 18+	Non- contrast Enhanced Multiparametric Renal Magnetic Resonance (*T*_1_, *T*_2_, *R*_2_*, ADC, perfusion, ASL)	Repeatability and reproducibility of multiple MRI biomarkers across time points (1–2 weeks)	Variation by age/gender, correlation with eGFR, acceptability, full dataset completeness

**Abbreviations:** CKD, chronic kidney disease; AKI, acute kidney injury; ADPKD, autosomal dominant polycystic kidney disease; MRI, magnetic resonance imaging; ASL, arterial spin labeling; DWI, diffusion weighted imaging; qMT-MRI, quantitative magnetization transfer magnetic resonance imaging; MRE, magnetic resonance elastography; PET, positron emission tomography; and US, ultrasound. *T*_1_/*T*_2_/*R*_2_*, MRI relaxation times (*T*_1_ and *T*_2_: longitudinal and transverse; *R*_2_*: inverse of *T*_2_*); ADC, apparent diffusion coefficient; PC-MRI, phase-contrast magnetic resonance imaging; and CEUS, contrast-enhanced ultrasound. MRA, magnetic resonance angiography; GFR, glomerular filtration rate; eGFR, estimated glomerular filtration rate; and CO₂, carbon dioxide.

## CONCLUSION

Kidney imaging is poised to revolutionize nephrology’s diagnostic and treatment paradigms. Whether it’s CKD, DKD, hypertensive nephrosclerosis, or kidney transplantation, quantitative imaging biomarkers provide direct, repeatable, and noninvasive insights into tissue-level pathology, including fibrosis, hypoxia, perfusion, lipid infiltration, and microvascular compromise, which traditional clinical measures are unable to detect. Customized therapy monitoring, focused treatments, and early risk assessment are made possible by these modalities. Advanced technologies such as H-scan, radiomics, PAI, and AI-driven analysis have markedly enhanced renal imaging. Collectively, hypoxia-sensitive BOLD, flow-sensitive ASL, micro-structural IVIM, stiffness-oriented SWE/MRE, native T1-mapping and vascular Doppler indices detect injury before traditional markers rise, stratify fast vs. slow progressors more accurately than eGFR or albuminuria alone and can be modulated by targeted therapy within days to months, laying the foundation for precision nephrology that individualizes risk prediction, therapeutic titration, and early assessment of drug efficacy. These technologies broaden their applications from diagnostics to prognostication and therapy optimization. The efficacy of imaging-based surrogate endpoints is limited by the need for standardization, integration into clinical workflows, regulatory acknowledgment, and longitudinal validation.

Despite advances in renal imaging, evidence that imaging-guided management directly slows CKD progression or reduces cardiovascular events is still limited. Future prospective studies are needed to establish the clinical and economic impact of imaging-guided strategies.

Future kidney imaging research should prioritize multicenter longitudinal trials, imaging-informed clinical trials, integration with multi-omics and digital health platforms, establishment of imaging thresholds associated with hard outcomes, and implementation science to enhance accessibility, cost-effectiveness, and guideline inclusion. Renal imaging is now a crucial component of precision nephrology, linking pathophysiology to tailored treatment plans thanks to recent developments.

## Data Availability

No new data created in this manuscript.
